# Supplementary material for: Systemic deficits in lipid homeostasis promote aging-associated impairments in B cell progenitor development
Source: GeroScience. 2025 Apr 15;47(4):5449–67. doi: 10.1007/s11357-025-01594-w (PMC12397465; doi:10.1007/s11357-025-01594-w)
Supplement: Supplementary file 4 — Supplementary file4 (PDF 869 KB) [file 11357_2025_1594_MOESM4_ESM.pdf]

Figure S1

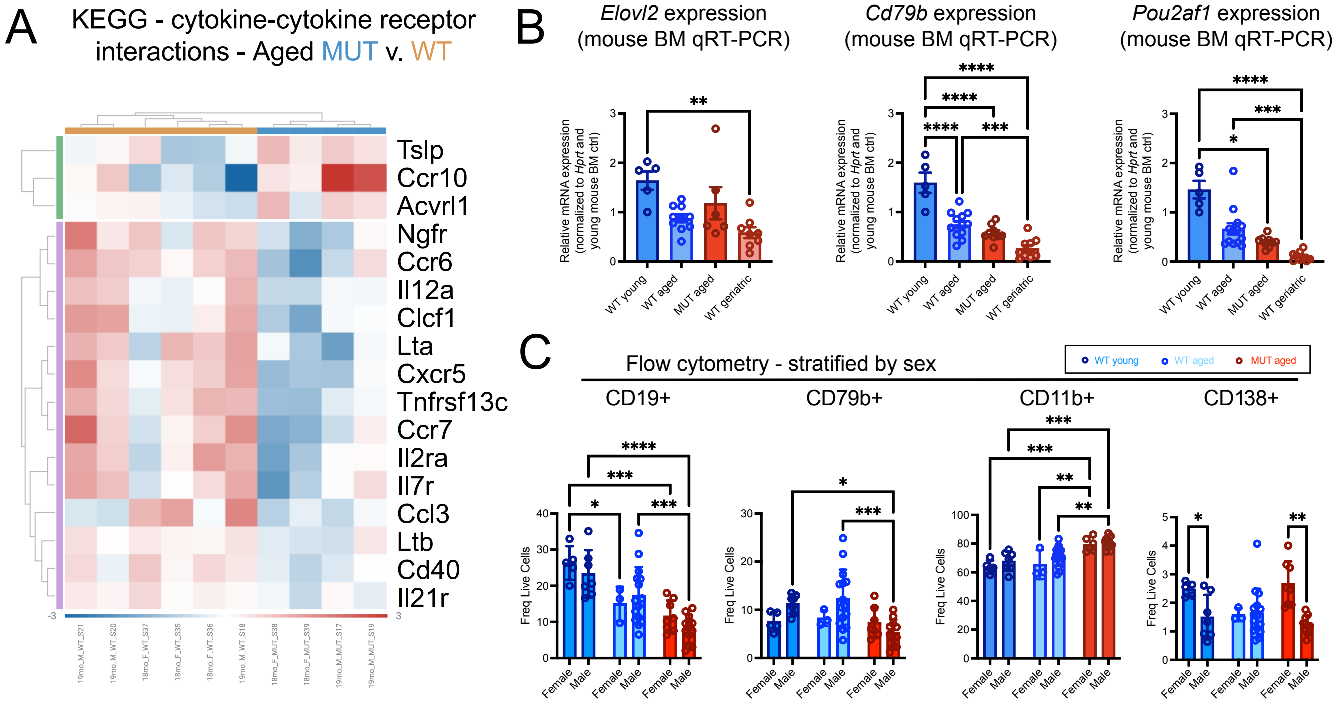

A
KEGG - cytokine-cytokine receptor interactions - Aged MUT v. WT
Tslp
Ccr10
Acvrl1
Ngfr
Ccr6
Il12a
Clcf1
Lta
Cxcr5
Tnfrsf13c
Ccr7
Il2ra
Il7r
Ccl3
Ltb
Cd40
Il21r
-3
3
B
Elovl2 expression (mouse BM qRT-PCR)
Cd79b expression (mouse BM qRT-PCR)
Pou2af1 expression (mouse BM qRT-PCR)
Relative mRNA expression (normalized to Hprt and young mouse BM ctrl)
WT young
WT aged
MUT aged
WT geriatric
C
Flow cytometry - stratified by sex
WT young
WT aged
MUT aged
CD19+
CD79b+
CD11b+
CD138+
Freq Live Cells
Female
Male

Figure S2

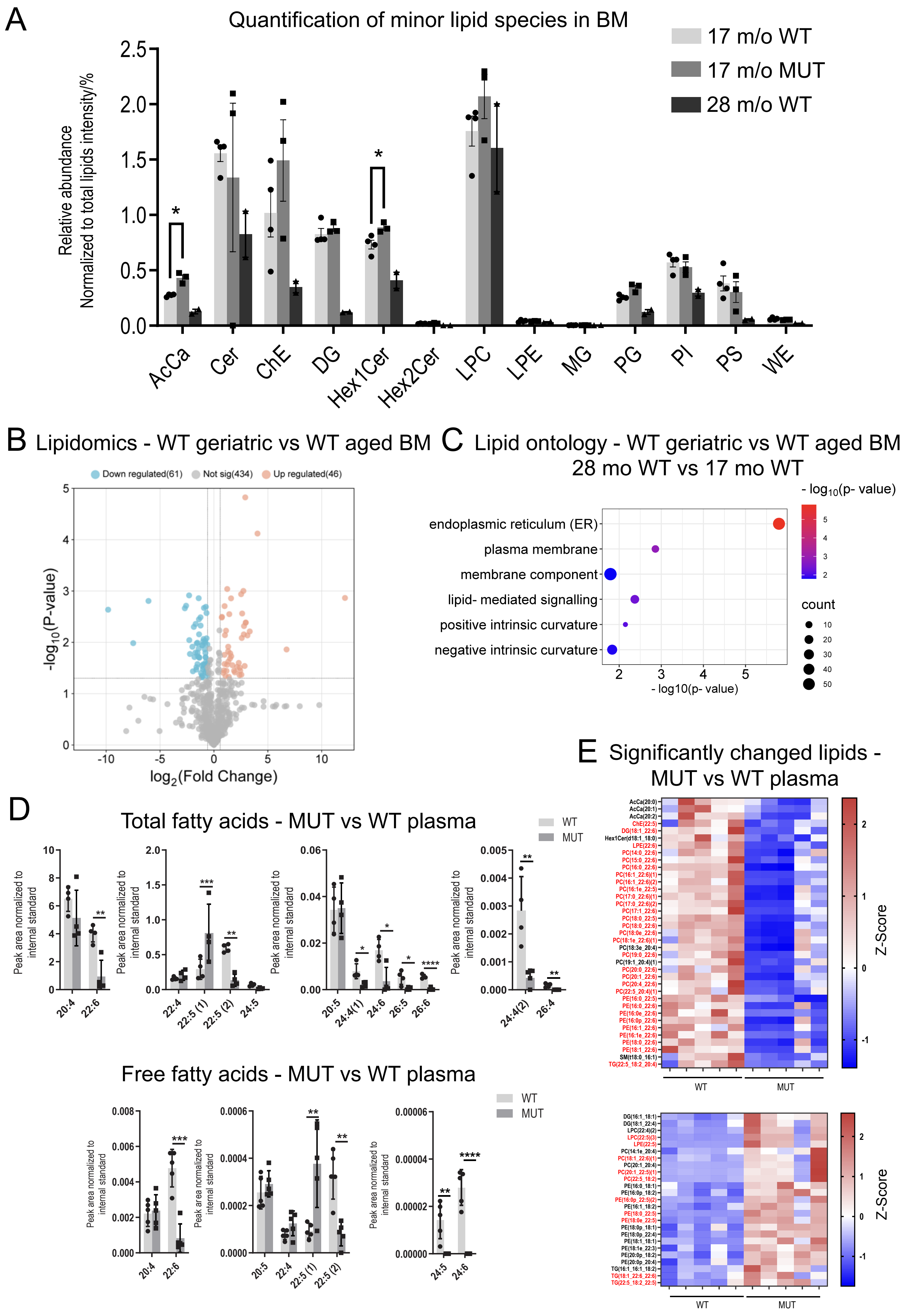
A
Quantification of minor lipid species in BM
17 m/o WT
17 m/o MUT
28 m/o WT
Relative abundance
Normalized to total lipids intensity/%
AcCa
Cer
ChE
DG
Hex1Cer
Hex2Cer
LPC
LPE
MG
PG
PI
PS
WE
B Lipidomics - WT geriatric vs WT aged BM
Down regulated(61)
Not sig(434)
Up regulated(46)
-log10(P-value)
log2(Fold Change)
C Lipid ontology - WT geriatric vs WT aged BM
28 mo WT vs 17 mo WT
endoplasmic reticulum (ER)
plasma membrane
membrane component
lipid- mediated signalling
positive intrinsic curvature
negative intrinsic curvature
- log10(p- value)
count
D
Total fatty acids - MUT vs WT plasma
WT
MUT
Peak area normalized to internal standard
Free fatty acids - MUT vs WT plasma
E Significantly changed lipids - MUT vs WT plasma
Z-Score
WT
MUT
