# Supplementary material for: Systemic deficits in lipid homeostasis promote aging-associated impairments in B cell progenitor development
Source: GeroScience. 2025 Apr 15;47(4):5449–67. doi: 10.1007/s11357-025-01594-w (PMC12397465; doi:10.1007/s11357-025-01594-w)
Supplement: Supplementary file 5 — Supplementary file5 (ZIP 897 KB) [file 11357_2025_1594_MOESM5_ESM.zip › Supp_Info-updated-Crews.pdf]

Supplementary Information

Supplementary Table Legends

Table S1 (related to Main Figure 1): Relative expression (log_2 normalized values) of significantly differentially expressed genes in aged MUT versus WT mouse bone marrow. Expression values of genes determined as significantly different (fold change >±1.25, $p<0.05$) in the Rosalind computational platform were exported and ranked in order of average relative expression in WT mice. When p-adj values were used (<0.05), a total of 24 genes were significantly differentially expressed, the majority of which represented B cell receptor signaling or related pathways. These values and samples from this gene list were also used for GSEA analysis to generate the heatmaps in Figure 1C and Figure S1A.

Table S2 (related to Main Figure 1): Top significantly enriched pathways among KEGG ortholog genes computed in GSEA.

Table S3 (related to Main Figure 1): List of transcription factor (TF) genes whose transcriptional targets overlap with genes that were differentially expressed in MUT aged versus WT mouse bone marrows. The ChIP-X Enrichment Analysis 3 (ChEA3) [28] tool was applied to evaluate all of the differentially expressed genes identified in Figure 1A and uncover potential transcription factor co-regulatory networks responsible for the gene expression changes observed in MUT aged versus WT mice.

Supplementary Figures and Legends

Figure S1 (related to Main Figures 1 and 2): Validation of multi-omics analyses and sex-stratified protein expression changes in the bone marrow of WT young, aged, geriatric, and ELOVL2 MUT mice. Total bone marrow cells from 18-19 month old aged WT versus ELOVL2 C234W MUT mice were analyzed by whole transcriptome RNA-sequencing (as in Figure 1) with expanded analyses in additional samples for qRT-PCR and flow cytometry.
A) Heatmap showing the relative expression levels of KEGG cytokine-cytokine receptor interactions pathway gene orthologs in aged MUT versus WT bone marrow samples.
B) qRT-PCR analysis of *Elovl2, Cd79b,* and *Pou2af1* gene expression in an expanded cohort of mouse bone marrow samples including WT young, MUT aged, WT aged and WT geriatric. For all panels, significance was determined using one-way ANOVA with parametric or non-parametric tests based on normality tests for each dataset (*p<0.05, **p<0.01, ***p<0.005, ****p<0.001; *Elovl2*, *Pou2af1*: Kruskal–Wallis with Dunn's multiple comparison post hoc test; *Cd79b*: One-Way ANOVA with Tukey's multiple comparison post hoc test).
C) Flow cytometry data from Main Figure 2 (A and C), stratified by sex. Since no geriatric female samples were available for these analyses, the geriatric condition was not plotted since n=4 were all males. For all panels, significance was determined using one-way ANOVA with parametric or non-parametric tests based on normality tests for each dataset (*p<0.05, **p<0.01, ***p<0.005, ****p<0.001; Cd19 and Cd79b: Two-Way ANOVA with Tukey's multiple comparison post hoc test; Cd11b: Two-Way ANOVA with Šidak's multiple comparison post hoc test; *p<0.05; Cd138: Mixed effect model with Dunn's multiple comparison post hoc test; *p<0.05).

Figure S1

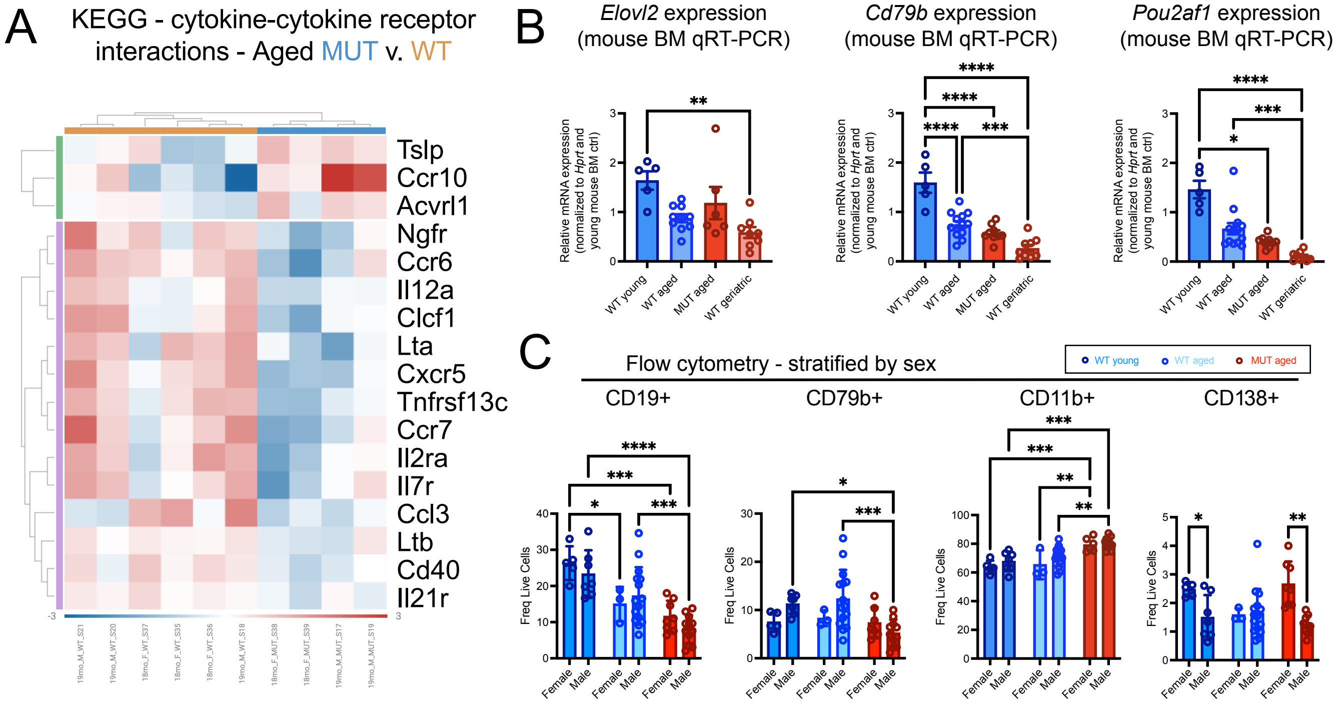

A
KEGG - cytokine-cytokine receptor interactions - Aged MUT v. WT
Tslp
Ccr10
Acvrl1
Ngfr
Ccr6
Il12a
Clcf1
Lta
Cxcr5
Tnfrsf13c
Ccr7
Il2ra
Il7r
Ccl3
Ltb
Cd40
Il21r
-3
3
B
Elovl2 expression (mouse BM qRT-PCR)
Cd79b expression (mouse BM qRT-PCR)
Pou2af1 expression (mouse BM qRT-PCR)
Relative mRNA expression (normalized to Hprt and young mouse BM ctrl)
WT young
WT aged
MUT aged
WT geriatric
C
Flow cytometry - stratified by sex
WT young
WT aged
MUT aged
CD19+
CD79b+
CD11b+
CD138+
Freq Live Cells
Female
Male

Figure S2 (related to Main Figure 3): Global lipidomic analyses of the bone marrow of WT aged, WT geriatric, and MUT aged mice and fatty acid and lipidomic analyses of plasma samples from aged MUT versus WT mice.
A) Relative abundance of minor lipid species compared among aged MUT vs. aged WT vs. geriatric WT. *$p<0.05$ compared to samples from WT aged (17 months old) mice.
B) Volcano Plot indicates significant differential expression between geriatric WT vs. aged WT bone marrow samples.
C) Lipid ontology analyses comparing WT geriatric versus aged bone marrow samples.
D) Total and free fatty acids analyses in aged (18 months old) MUT vs WT plasma samples (*$p<0.05$ compared to WT aged plasma samples).
E) Heatmaps showing differential abundance (fold change >1.5, $p<0.05$) of lipid species in aged MUT vs WT plasma (upper panel shows significantly downregulated lipids and lower panel shows significantly upregulated lipids in aged MUT plasma).

Figure S2

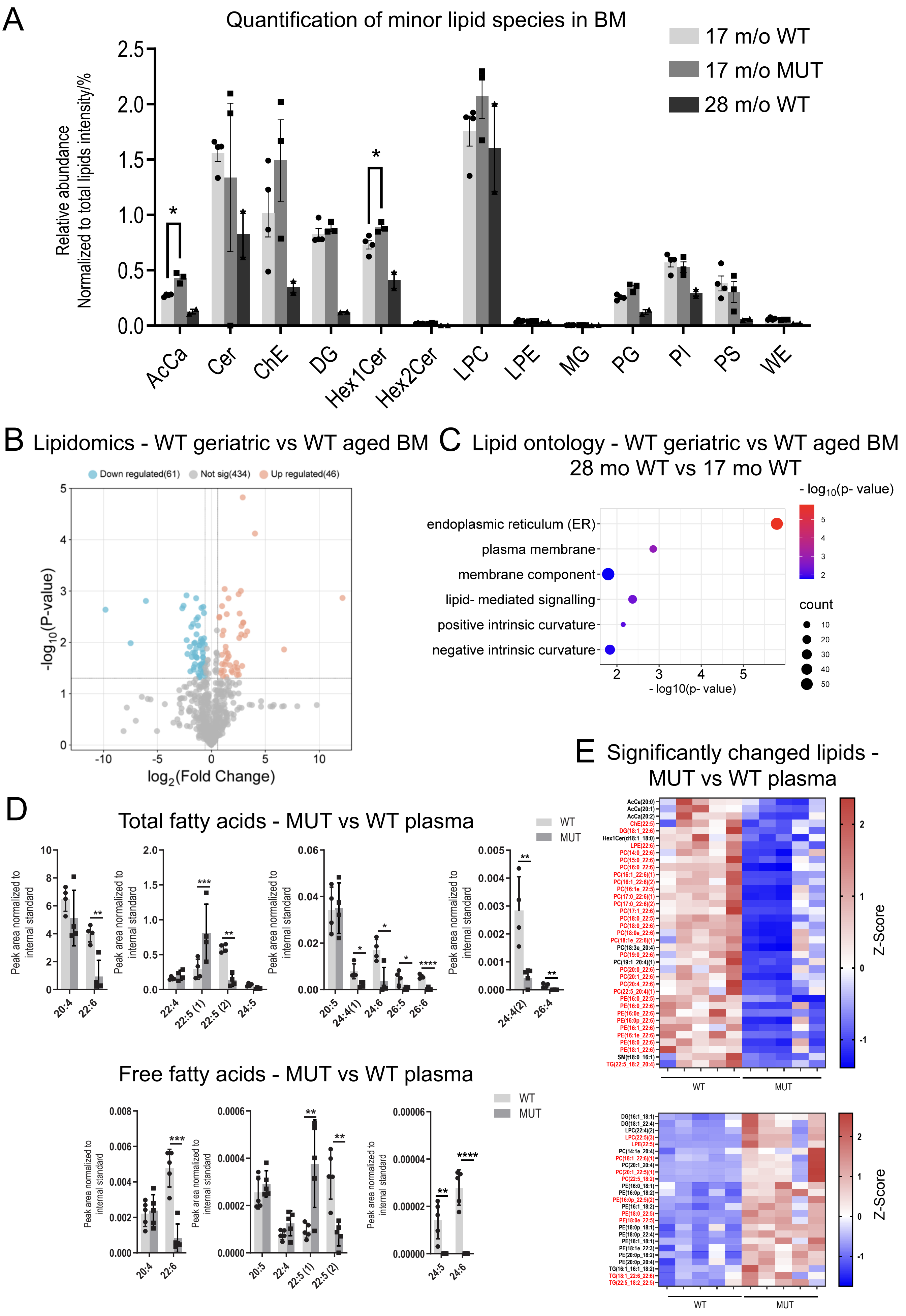

A
Quantification of minor lipid species in BM
17 m/o WT
17 m/o MUT
28 m/o WT
Relative abundance
Normalized to total lipids intensity/%
AcCa
Cer
ChE
DG
Hex1Cer
Hex2Cer
LPC
LPE
MG
PG
PI
PS
WE
B Lipidomics - WT geriatric vs WT aged BM
Down regulated(61)
Not sig(434)
Up regulated(46)
-log10(P-value)
log2(Fold Change)
C Lipid ontology - WT geriatric vs WT aged BM
28 mo WT vs 17 mo WT
endoplasmic reticulum (ER)
plasma membrane
membrane component
lipid- mediated signalling
positive intrinsic curvature
negative intrinsic curvature
- log10(p- value)
count
D
Total fatty acids - MUT vs WT plasma
WT
MUT
Peak area normalized to internal standard
20:4
22:6
22:4
22:5 (1)
22:5 (2)
24:5
20:5
24:4(1)
24:6
26:5
26:6
24:4(2)
26:4
Free fatty acids - MUT vs WT plasma
E Significantly changed lipids - MUT vs WT plasma
Z-Score
WT
MUT
